# Defect-selective-etched porous GaN as a buffer layer for high efficiency InGaN/GaN light-emitting diodes

**DOI:** 10.1371/journal.pone.0277667

**Published:** 2022-11-17

**Authors:** Ah Hyun Park, Seungjae Baek, Young Won Kim, S. Chandramohan, Eun-Kyung Suh, Tae Hoon Seo

**Affiliations:** 1 R&D Center, Marine Equipment Technology Solutions, Busan, Republic of Korea; 2 Maritime ICT R&D Center, Korea Institute of Ocean Science and Technology, Busan, Republic of Korea; 3 Green Energy & Nano Technology R&D Group, Korea Institute of Industrial Technology, Gwangju, Republic of Korea; 4 Department of Physics and Nanotechnology, SRM Institute of Science and Technology, Kattankulathur, Tamil Nadu, India; 5 School of Semiconductor and Chemical Engineering, Semiconductor Physics Research Center, Jeonbuk National University, Jeonju, Republic of Korea; Nanjing Normal University, CHINA

## Abstract

Substrate-induced biaxial compressive stress and threading dislocations (TDs) have been recognized to severely impair the performance, stability, and reliability of InGaN/GaN light-emitting diodes (LEDs) for quite some time. In this study, a defect-selective-etched (DSE) porous GaN layer is fabricated employing electro-chemical etching and applied as a buffer layer for the development of InGaN/GaN LEDs with high quantum efficiency. Based on the analysis of photoluminescence and micro-Raman spectra, it has been revealed that the overgrown GaN epilayer on the DSE porous GaN has a relatively low TDs and relaxation of compressive stress in comparison to the conventional GaN epilayer. The remarkable improvement in the internal quantum efficiency of the InGaN/GaN LEDs is directly attributable to the strong radiative recombination in InGaN/GaN multi-quantum-wells caused by stress relaxation and TDs annihilation. Our findings indicate that the use of DSE porous GaN as a buffer layer may be a viable approach for producing crystalline GaN epilayers and high-performance LEDs.

## Introduction

Gallium nitride (GaN)-based light-emitting diodes (LEDs) have been widely used over the past few decades for a plethora of visible light applications due to technological advancement of the field in terms of device efficiency, durability, and stability [1–5]. While the latter two qualities are to a large extent inherent to the materials properties, the device efficiency, the sum of internal and external quantum efficiencies, is principally decided by the structural and optical quality of the active layers and the device configuration. The most ideal way to achieve a maximum internal quantum efficiency (IQE) is to grow the active layers on a native GaN substrate. However, due to the high cost of the native substrate GaN-based LEDs are usually grown on a foreign substrate such as c-plane sapphire, SiC or Si. Sapphire has a particular advantage for being highly stable at around the GaN growth temperatures, and its easily affordable price and manufacturing readiness level add further merits to make it the most common substrate of use in the GaN-based LED industry. However, the direct growth of GaN-based LEDs on sapphire leads to reduced degree of crystallinity. This is due to the fact that the mismatches in lattice constant (and thermal expansion coefficient) between the GaN and the sapphire accommodate a biaxial compressive strain during the heteroepitaxy of GaN. As a result, threading dislocations (TDs) emanate from the interface and further propagates into subsequently grown InGaN active layers [6–8]. Accordingly, the quantum efficiency degradation of GaN-based LEDs is inevitable as TDs are known to act as non-radiative recombination centers [9, 10].

In recent years, a variety of methods have been proposed to improve the quantum efficiency of the GaN-based LEDs. Epitaxial lateral overgrowth (ELO) is the most widely used method for the growth of high crystal quality GaN epilayer [11–14], wherein a patterned interlayer (such as SiO_2_ or SiN_x_) or a patterned substrate is the key requirements in order to facilitate the lateral overgrowth. Recently, nanoscale-materials-based augmented ELO has gained considerable attention due to its potential for more efficient TD annihilation and stress relaxation [15–17]. While ELO is still considered as an effective approach for the heteroepitaxy of GaN LEDs, the complexity of manufacturing process and associated cost increase holds this method back from being a choice for industry scale production.

Meanwhile, wet-chemical etching (WCE) has emerged as a potential method in nitride semiconductor industry, mostly for patterning of the layer, but at the same time it remains to be a key surface processing route for effectively controlling the lateral overgrowth [18–21]. Besides its advantage as a simple process, WCE can etch defect sites selectively in GaN epilayers under the use of specific etchants such as phosphoric acid (H_3_PO_4_), molten potassium hydroxide (KOH), and mixed solution of H_3_PO_4_/H_2_SO_4_ [22–24]. However, the highly anisotropic etching nature of the defects in GaN epilayer can cause merging of etch pits, which in turn can increase the etch pattern size. In contrast, electro-chemical etching (ECE) as a well controllable approach is gaining considerable interest within the nitride device community in recent times [25–27]. For instance, ECE has the potential for selective lateral etching of certain sacrificial nitride layers in the realization of thin film flip-chip LEDs [26], freestanding devices or III-nitride membranes [27–29], etc. Recently, it has been reported that the GaN-based LED membranes with a complete device structure from the Si substrate could be achieved by ECE of highly conductive AlN/Si interface [30]. Also, N. Fiuczeka *et al*. demonstrated well-controlled ECE of p-type GaN under a constant bias using a tunnel junction between the p- and n-GaN layers to inject the hole carriers for etching to occur at the p-GaN/solution interface [31]. The ECE is not only simple, but also a very efficient and precise fabrication approach for generating porous GaN layer [25, 32, 33]. In this study, we employed an ECE approach standardized by our group [34], in order to maintain the etch pattern size in the nanoscale regime. In this process, the GaN epilayer undergoes selective etching at most of the defect sites and regions of highest conductivity, leading to a porous GaN layer with etch pit density (EPD) almost comparable to the density of dislocations [34]. According to our earlier findings based on transmission electron microscopy observations, the dislocation lines propagating further during the overgrowth step of GaN epitaxy on such defect-selective-etched (DSE) GaN tend to bend laterally and annihilate. Using the DSE porous GaN layer as a template, herein we demonstrate the growth of high quality GaN epilayer and its application to fabricate high efficiency GaN-based LEDs.

## Materials and methods

### Preparation of DSE porous GaN layer and overgrown GaN epilayer on the DSE porous GaN

The GaN epilayers and the multiple-quantum well (MQW) LED structures are grown on a (0001) c-plane sapphire substrate by metal-organic chemical vapor deposition (MOCVD) system. Prior to the GaN growth, the substrate is subjected to a thermal cleaning step, followed by a 25 nm-thick buffer GaN layer is grown at 560°C for 120 s at a growth pressure of 400 mbar. Subsequently, growth of a 2 *μ*m-thick un-doped GaN epilayer is carried out at 1100°C for 120 min. After this step, to fabricate the DSE porous GaN layer, the sample is taken out from the MOCVD reactor and subjected to the ECE in 0.3 M oxalic acid solution at a constant current of 1 mA for 20 min at 5°C, using carbon plate as a cathode and un-doped GaN epilayer as a working electrode. More details of ECE of GaN can be found in Ref. 34. This is the optimal condition for obtaining uniform and a high density of porous etch pits (refer S1 Fig in the supplementary information). Then, the sample after thorough cleaning is loaded back into the MOCVD reactor and a 2 *μ*m-thick un-doped GaN epilayer is overgrown onto the DSE porous GaN layer (which now serves as a buffer layer) at 1100°C for 120 min under a growth pressure of 400 mbar.

### Growth of LED structures

InGaN/GaN MQW LED is realized on this substrate through the following growth steps. First, a 2 *μ*m-thick *n*-type GaN epilayer is grown at 1100°C for 120 min at a pressure of 400 mbar. Thereafter, five pairs of InGaN quantum wells and GaN barriers are grown successively at 780°C and 870°C, respectively. Finally, a 150 nm-thick *p*-type GaN epilayer is grown at 980°C and thermal activation of the *p*-type GaN epilayer is carried out at 940°C for 40 s under ambient nitrogen by rapid thermal annealing. In summary, the InGaN/GaN MQW LED consisted of a DSE porous GaN buffer layer, an un-doped GaN epilayer, a 2 *μ*m-thick Si-doped *n*-type GaN epilayer, five pairs of In_0.03_Ga_0.97_N/GaN MQWs, and a 150 nm-thick Mg-doped *p*-type GaN epilayer. For comparison, a reference InGaN/GaN MQW LED epilayer is grown on a conventional un-doped GaN epilayer (in place of DSE porous GaN buffer layer) in the same growth run to ensure reliability in comparison between the two devices.

### Fabrication of LEDs

**Fig 1:**
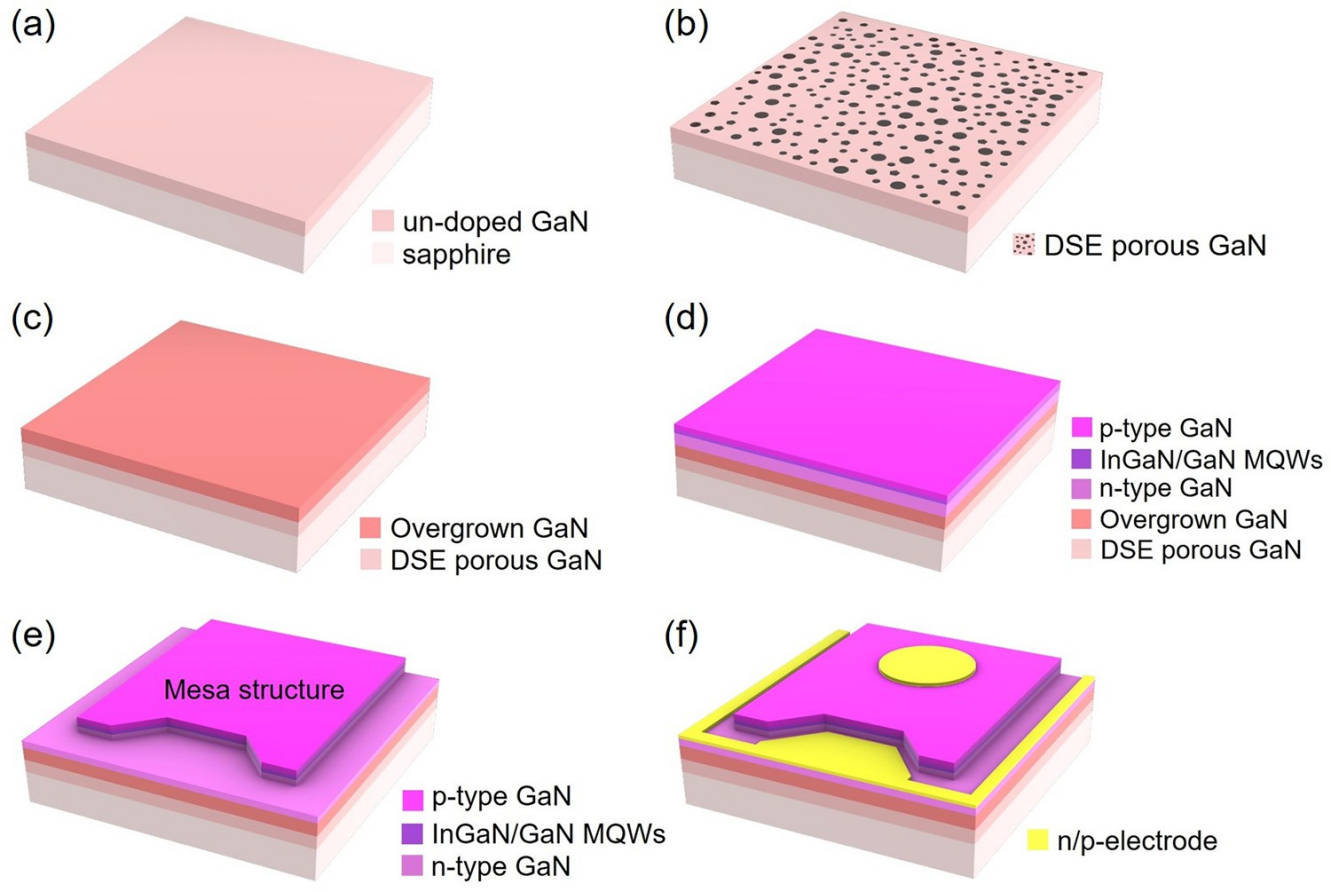
Schematic diagram. Fabrication process of the InGaN/GaN LEDs on a DSE porous GaN layer.

For the fabrication of isolated LED arrays on a wafer-scale, the InGaN/GaN MQW LED epi-wafer is etched until the *n*-type GaN epilayer is exposed for *n*-electrode formation using inductively coupled plasma reactive ion etching in conjunction with photolithography steps. Subsequently, a 200 nm-thick indium tin oxide (ITO) is deposited selectively on the *p*-GaN layer as a transparent and current spreading layer by electron beam evaporation. Finally, Cr (50 nm)/Au (250 nm) metals for the *p*- and the *n*-electrode are deposited onto both the ITO and the *n*-type GaN epilayer. A schematic diagram of the fabricated InGaN/GaN MQW LED is shown in Fig 1.

### Characterization

The surface and cross-sectional morphology of the DSE porous GaN buffer layer and the overgrown un-doped GaN epilayer are analyzed by field-emission scanning electron microscopy (FESEM) and atomic force microscope (AFM). Raman and photoluminescence (PL) spectroscopic analyses are performed to investigate the crystalline quality and the residual stress of un-doped GaN epilayer overgrown on DSE porous GaN buffer layer and conventional GaN epilayer. The 632.8 nm-line of a He-Ne laser and the 325 nm-line of a He-Cd laser are used, respectively, as the excitation sources for Raman and PL spectral data collection. For the analysis of the radiative recombination efficiency of the MQWs, temperature-dependent and power-dependent PL measurements are also performed. Current-voltage (I-V), electroluminescence (EL), and light output power measurements are carried out on unpackaged LED chips using a probe station equipped with a parameter analyzer and a silicon photo detector.

## Results and discussion

**Fig 2:**
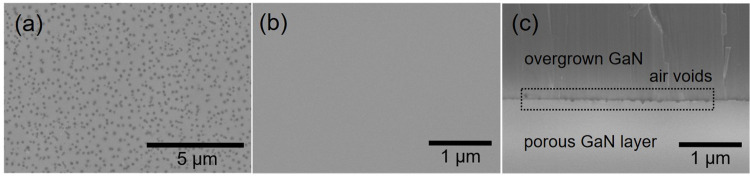
Characteristics of surface and cross-sectional morphology of overgrown GaN on DSE porous GaN. SEM images of (a) DSE porous GaN layer, (b) overgrown GaN epilayer on the DSE porous GaN layer, and (c) their interface.

The morphologies of the GaN layer subjected to ECE and the overgrown GaN epilayer are studied by FESEM imaging. One can see from Fig 2(A) that the porous etch pits are randomly and densely formed in the GaN layer as a result of ECE. Since defect sites that exist in GaN layer are chemically weak and have many dangling bonds, they become reactive in acid catalysis and hence easily get etched [34–36]. The average diameter of the porous etch pits is estimated to be approximately 100 nm and the etch pit density (EPD) is estimated to be approximately 9.1×10^8^ cm^-2^, which is close to the typical TD density in the GaN layer [8]. Fig 2(B) shows the surface morphology of the GaN epilayer overgrown on the DSE porous GaN buffer layer. It is well-known that the ELO often leave behind a trace during the coalescence process [37]. In our method, the overgrowth of the GaN epilayer on the DSE porous GaN buffer layer is estimated to favor coalescence process with a vanishing boundary, thus allowing for a flat overgrown GaN epilayer. This is highly probable as the nanoscale etch pits have the tendency to facilitates nanoscale ELO during the overgrowth on the DSE porous GaN buffer layer [34]. In addition, at the interface between the overgrown GaN epilayer and the DSE porous GaN buffer layer, air voids with a size distribution in the 100–150 nm range are observed, as shown in Fig 2(C). These air voids can serve as distributed Bragg reflector in enhancing the light extraction efficiency (LEE) of LEDs. The TD annihilation around the air voids can result a highly crystalline GaN epilayer with reduced defect density [17, 38, 39].

**Fig 3:**
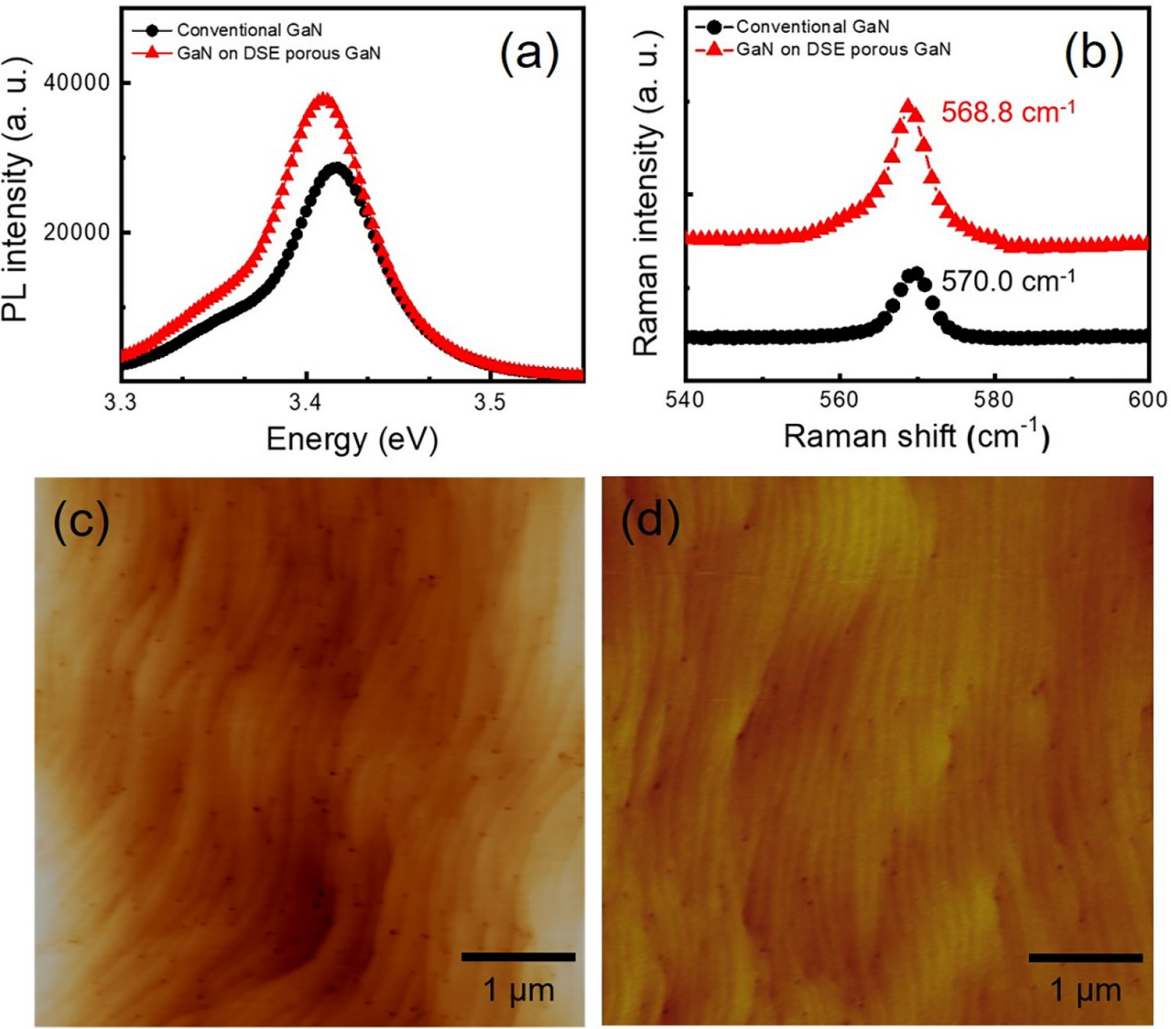
Evaluation of the effect of DSE porous GaN on the crystal quality and residual stress. (a) PL and (b) micro-Raman spectra of the conventional GaN and GaN epilayer overgrown on DSE porous GaN layer. AFM surface topography of (c) conventional GaN and (d) GaN epilayer overgrown on DSE porous GaN layer.

To study the crystal quality and the internal residual stress in GaN epilayer overgrown on the DSE porous GaN buffer layer, we carried out PL and micro-Raman measurements. Fig 3(A) shows the PL spectra of the conventional GaN epilayer and the GaN epilayer overgrown on the DSE porous GaN buffer layer. The PL spectrum is dominated by near band-edge (NBE) luminescence at 3.409 and 3.420 eV, respectively, for the GaN epilayer with and without the DSE porous GaN buffer layer. This shows that the NBE emission peak of the GaN epilayer overgrown on the DSE porous GaN buffer layer is red-shifted by 11 meV, relative to the peak position of conventional GaN epilayer. Considering the fact that biaxial stress can induce about 23 meV/GPa shift in NBE emission peak, the estimated 11 meV red shift corresponds to a stress relaxation of 0.48 GPa [40]. Moreover, it is well known that the NBE intensity is sensitive to the defect density [41]. The PL intensity of the GaN epilayer overgrown on the DSE porous GaN buffer layer is increased, by approximately 25%, compared to that of the conventional GaN epilayer. This can be attributed to the improvement in crystalline quality of the GaN epilayer caused by the reduction in TDs and efficient stress relaxation. Fig 3(B) shows the micro-Raman spectra for the E_2_ (high) phonon mode in GaN. In general, the GaN epilayers grown on sapphire substrate accommodate a compressive biaxial stress due to difference in the thermal expansion coefficient between the sapphire and the GaN layer during the cool down step in the growth process. In our study, the conventional GaN epilayer grown on sapphire is found to be compressively strained, as the E_2_ (high) phonon frequency is blue-shifted by 2.4 cm^-1^, from the reported value (567.6 cm^-1^) of bulk stress-free GaN epilayer [42]. For the GaN epilayer overgrown on DSE porous GaN buffer layer, the E_2_ (high) phonon frequency is observed at 568.8 cm^-1^, which suggests a red-shift of the peak by 1.2 cm^-1^ relative to the position of the conventional GaN epilayer, verifying relaxation of compressive stress in the GaN epilayer overgrown on DSE porous GaN buffer layer. The degree of stress relaxation can be calculated from the empirical relation, Δ*ω* = *K.σ_xx_*, where Δ*ω* is the difference in the measured E_2_ (high) phonon frequency from the bulk stress-free GaN epilayer value (567.6 cm^-1^), and *K* is the biaxial stress coefficient, 2.56 cm^-1^/GPa [43]. Accordingly, the biaxial stress values are estimated to be 0.94 and 0.47 GPa for conventional GaN epilayer and the GaN epilayer overgrown on the DSE porous GaN buffer layer, respectively. It is evident that the GaN epilayer overgrown on the DSE porous GaN buffer layer benefits from low internal residual stress due to edge dislocations inclination and annihilation induced by the ELO process [16]. Furthermore, the full width at half maximum of the E_2_ (high) phonon mode is estimated to be 5.21 and 5.10 cm^-1^ respectively, for the GaN epilayers on conventional and DSE porous GaN buffer layer.

To give a further insight into the structural quality of the grown GaN epilayer, we carried out AFM imaging of the surface, as shown in Fig 3(C) and 3(D). This method allows us to easily analyze the differences in the sample quality. We observe a decrease in the root mean square (RMS) roughness from 0.55 nm to 0.34 nm, for the overgrown GaN on the DSE porous GaN buffer layer. In addition, the EPD, which on an average will be equal to the density of dislocations in GaN, is estimated to be 8.6×10^8^ cm^-2^ and 2.3×10^8^ cm^-2^, respectively, for the conventional GaN epilayer and the GaN epilayer overgrown on the DSE porous GaN buffer layer. It may be noted that the EPD value estimated from the SEM image after ECE process is comparable to the EPD value estimated from AFM image, indicating that the defect sites in the GaN layer are etched in acid catalysis. The reduced RMS and EPD values in GaN epilayer overgrown on the DSE porous GaN buffer layer suggests a significant suppression of TDs propagation. These results signify that high-quality GaN epilayers can be obtained on DSE porous GaN buffer layer.

**Fig 4:**
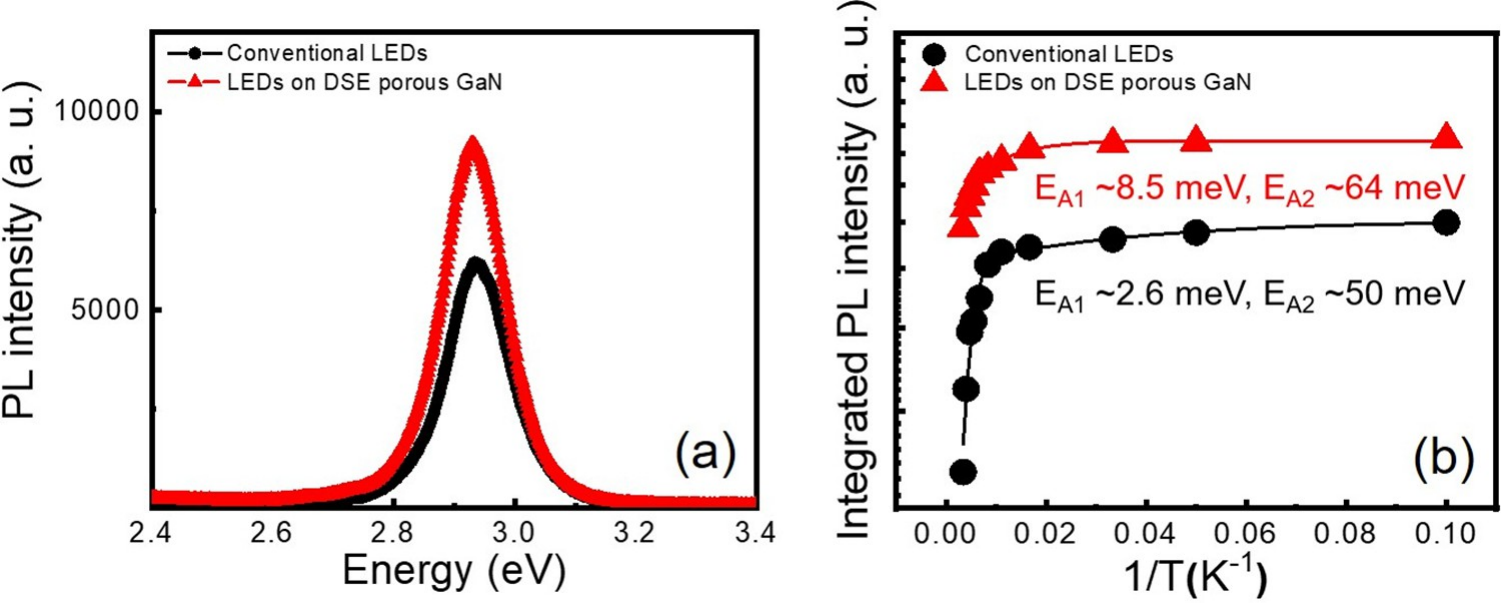
Optical property of the LEDs fabricated using DSE porous GaN as a buffer layer. (a) PL spectra and (b) Arrhenius plots of the integrated PL intensities for the two LEDs studied.

Fig 4(A) compares the PL emission spectra of MQWs (or in other words LED epilayers) grown on the DSE porous GaN buffer layer and conventional GaN layer. The emission energies in both samples are nearly same with a peak maximum occurring at 2.93 eV. But, the PL intensity of the LED epilayers on the DSE porous GaN buffer layer is increased by 32% compared to that of the conventional LED epilayers. The observed PL enhancement is due to the reduced defect density by TD annihilation and improved radiative recombination by stress relaxation within the MQWs active region. In order to further validate the above results, the activation energy is estimated using the temperature-dependent PL measurements. Fig 4(B) shows the temperature dependence of normalized integrated PL intensity, which can be fitted to the following Arrhenius equation, $I(T)=\frac{1}{1+C_{1}\;\exp(\frac{-E_{A1}}{K_{B}T})+C_{2}\;\exp\left(\frac{-E_{A2}}{K_{B}T}\right)}$, where *I(T)* is the normalized integrated PL intensity at assigned temperature *T*, *C*_*1*_ and *C*_*2*_ are constants related to the density of nonradiative recombination center, *k*_B_ is the Boltzmann’s constant, *E*_*A1*_ and *E*_*A2*_ are the activation energies concerning the nonradiative recombination centers at low and high temperature regions, respectively. The *E*_*A1*_ represents the localized exciton binding energy whereas the *E*_*A2*_ is associated with the potential barrier between the localized potential for thermal activation confined carriers in MQWs active region [34]. By fitting the experimental data using the above Arrhenius equation, the values of *E*_*A1*_ and *E*_*A2*_ are estimated to be, respectively, 2.6 and 50 meV for the conventional LED epilayers and 8.5 and 64 meV for the LED epilayers with the DSE porous GaN buffer layer. The observed enhancement in activation energy suggests a stronger confinement of carriers within the LED epilayers grown on the DSE porous GaN buffer layer. This can be taken as direct experimental evidence of reduced TD density and efficient stress relaxation within the MQWs active region. The internal quantum efficiency (IQE) is yet another crucial parameter that determines the performance of the LEDs. It is usually defined as the ratio of the integrated PL intensity at 10 K to that at 300 K, assuming that the nonradiative recombination is negligible at 10 K [44, 45]. The IQE values are estimated to be 32 and 60% for the conventional LED epilayers and LED epilayers grown on the DSE porous GaN buffer layer, respectively. The higher activation energy and the enhanced IQE strongly supports the improved radiative recombination in MQWs grown on defect free, stress relaxed GaN buffer layer, which can offer a better overlapping between the electron and hole wavefunctions in InGaN/GaN MQWs [16, 46].

**Fig 5:**
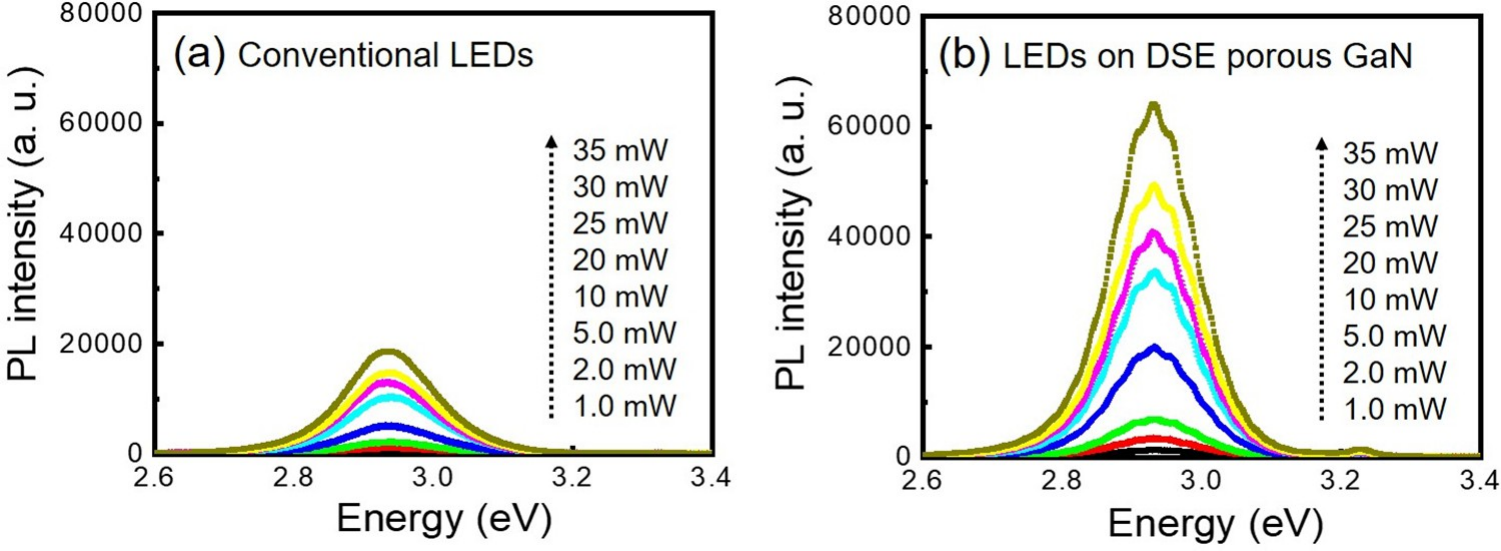
Stress relaxation within the MQWs grown on DSE porous GaN. Excitation-power dependent PL spectra of (a) conventional LEDs and (b) LEDs on DSE porous GaN layer.

To further support the result of the stress relaxation within the MQWs, the power-dependent PL spectra are recorded for laser power in the range from 1–35 mW and the results are shown in Fig 5. The PL peak position of the LED epilayers on the DSE porous GaN buffer layer at 1.0 mW excitation laser power is found to be blue-shifted by 4 meV relative to the value measured at 35 mW, while for the conventional LED epilayers the estimated blue-shift in the peak position is 11 meV. It is well known that piezoelectric fields induced by the residual strain in the MQWs can lead to quantum-confined Stark effect (QCSE) (the stronger the QCSE is, higher the blue shift of the PL peak) and reduce the radiative recombination efficiency. Therefore, the emission energy with increasing excitation power causes a blue-shift due to the band filling and screening effect of carriers [47]. The observed small blue shift (4 meV) in the LED epilayers on the DSE porous GaN buffer layer signifies a relatively weak QCSE, which suggests that the residual compressive stress is well relaxed in the MQWs.

**Fig 6:**
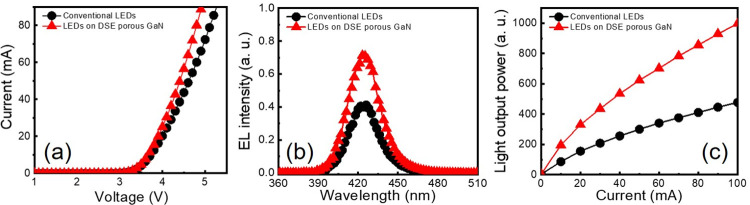
Opto-electric properties of the LEDs with DSE porous GaN. (a) I-V characteristics, (b) EL spectra, and (c) light output power of the two LEDs investigated in this study. The EL spectra are recorded at an applied current of 20 mA.

To understand the real potential of the DSE porous GaN buffer layer in LEDs, the opto-electrical properties of the LEDs are investigated. Fig 6(A) shows the measured I-V characteristics of respective LEDs at an injection current of 20 mA. The forward voltage is estimated to be 3.9 and 3.7 V, respectively, for the conventional and the DSE porous GaN buffer layer incorporated LEDs. The reduced forward voltage observed for the later LED can be attributed to the decrease in the reverse leakage current (S3 Fig of the supporting information), which scale with the amount of TD density. In general, TDs in LED epilayers serve as current leakage paths and hence act as non-radiative recombination centers. Ideally, *n*/*p*-GaN epilayers with low TDs provide efficient current diffusion pathways which then deliver the current to active regions with uniform current densities. Therefore, the reduced forward voltage and increased slope of the I-V curve of the LEDs grown on DSE porous GaN buffer layer can be considered an indication of suppression of TDs. From Fig 6(B) and 6(C), one can observe that the EL intensity and light output power of the LEDs with DSE porous GaN buffer layer are significantly enhanced by 45% and 53%, respectively, compared to conventional LEDs performance, at an injection current of 20 mA. Of particular point of interest is the different enhancement factor for the light output power and the IQE. This can be attributed to the fact that the presence of air voids within the epilayer structure provide a maximum index contrast which efficiently scatter and reflect the light to increase the LEE [48, 49].

## Conclusion

In summary, electro-chemical etching provided a way for defect-selective etching of GaN, which in turn allowed us to fabricate a porous GaN buffer layer for stress-relaxed growth of high quality InGaN/GaN LED epilayer structure. The photoluminescence and micro-Raman spectroscopy provided evidences for the relaxation of the internal residual stress and reduction in threading dislocations within the active layers of the LEDs grown on the porous GaN, which served as an effective buffer layer. A significant enhancement in the opto-electrical performance of the LEDs on the defect-selective etched porous GaN buffer layer is demonstrated. Our findings show that the defect-selective etched porous GaN buffer layer can be a better alternative to complex and expensive pattern formation strategies being used for epitaxial lateral overgrowth in producing high efficiency InGaN/GaN LEDs.

## Supporting information

S1 Fig Surface morphology of the DSE porous GaN. To obtain uniform porous etch pits, we carried out ECE for different durations. S1 Fig shows the surface morphology of the DSE porous GaN buffer layer for different etching durations; 5, 20, and 40 min. The diameter and density of porous etch pits are strongly dependent on the etching time. In case of etching time of 5 min, the porous etch pits are not perfectly etched. When the etching time increased, the diameter of the porous etch pits increased due to merging of adjacent etch pits with one another. As a result, the etch pit density decreases with increasing etch duration. Based on the results, etching time of 20 min is identified to be the optimal condition for obtaining uniform and a high density of porous etch pits.
(TIF)

S2 Fig X-ray diffraction measurement of MQWs grown on DSE porous GaN. S2 Fig shows the HRXRD spectrum of (002) reflection for omega-2theta scan of InGaN/GaN MQWs investigated in this work. The peak originating from GaN (002) plane, and the higher order satellite peaks (from -3 to 2) arising from the periodicity of the MQWs are clearly observed. The distinct and periodic satellite peaks indicate that the interface between the InGaN and GaN is very abrupt and the crystal quality is high. The indium composition in InGaN active layer is estimated to be approximately 3% by fitting the rocking curve.
(TIF)

S3 Fig Reverse bias voltage-current curves for two LEDs. S3 Fig shows the leakage currents of LEDs with and without DSE porous GaN, respectively. The leakage current is estimated to be 2 nA and 80 nA, respectively, for the LEDs grown on DSE porous GaN and conventional GaN, at a reverse voltage of -5V. The observed low reverse bias current can be attributed to the elimination of dislocations by DSE porous GaN.
(TIF)
