# Supplementary figures and images for: Defect-selective-etched porous GaN as a buffer layer for high efficiency InGaN/GaN light-emitting diodes
Source: PLoS One. 2022 Nov 17;17(11):e0277667. doi: 10.1371/journal.pone.0277667 (PMC9671350; doi:10.1371/journal.pone.0277667)

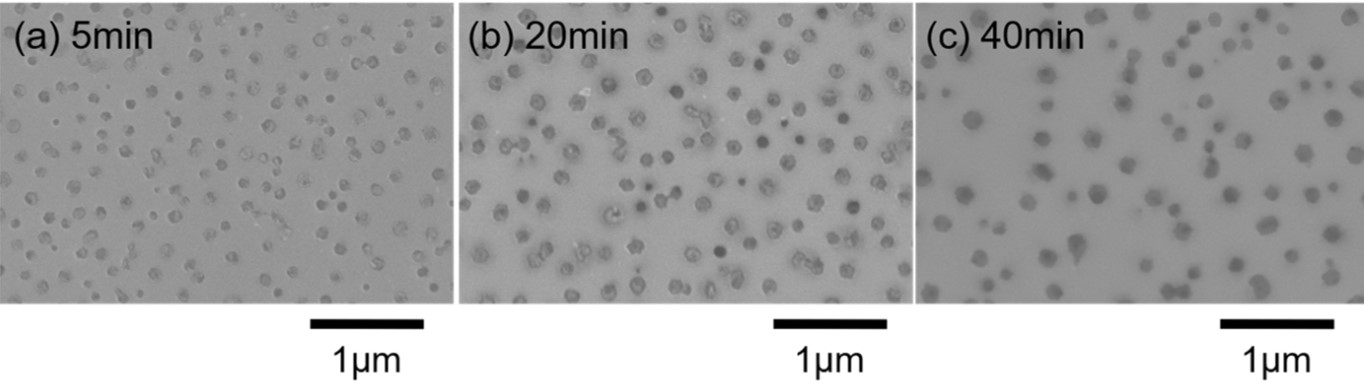

Supplement: S1 Fig — To obtain uniform porous etch pits, we carried out ECE for different durations. S1 Fig shows the surface morphology of the DSE porous GaN buffer layer for different etching durations; 5, 20, and 40 min. The diameter and density of porous etch pits are strongly dependent on the etching time. In case of etching time of 5 min, the porous etch pits are not perfectly etched. When the etching time increased, the diameter of the porous etch pits increased due to merging of adjacent etch pits with one another. As a result, the etch pit density decreases with increasing etch duration. Based on the results, etching time of 20 min is identified to be the optimal condition for obtaining uniform and a high density of porous etch pits. (TIF) [file pone.0277667.s001.tif]

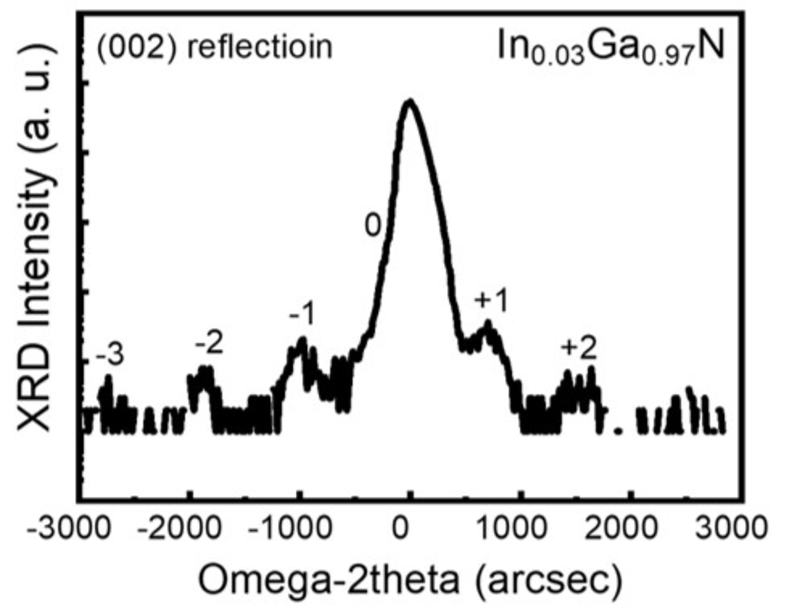

Supplement: S2 Fig — shows the HRXRD spectrum of (002) reflection for omega-2theta scan of InGaN/GaN MQWs investigated in this work. The peak originating from GaN (002) plane, and the higher order satellite peaks (from -3 to 2) arising from the periodicity of the MQWs are clearly observed. The distinct and periodic satellite peaks indicate that the interface between the InGaN and GaN is very abrupt and the crystal quality is high. The indium composition in InGaN active layer is estimated to be approximately 3% by fitting the rocking curve. (TIF) [file pone.0277667.s002.tif]

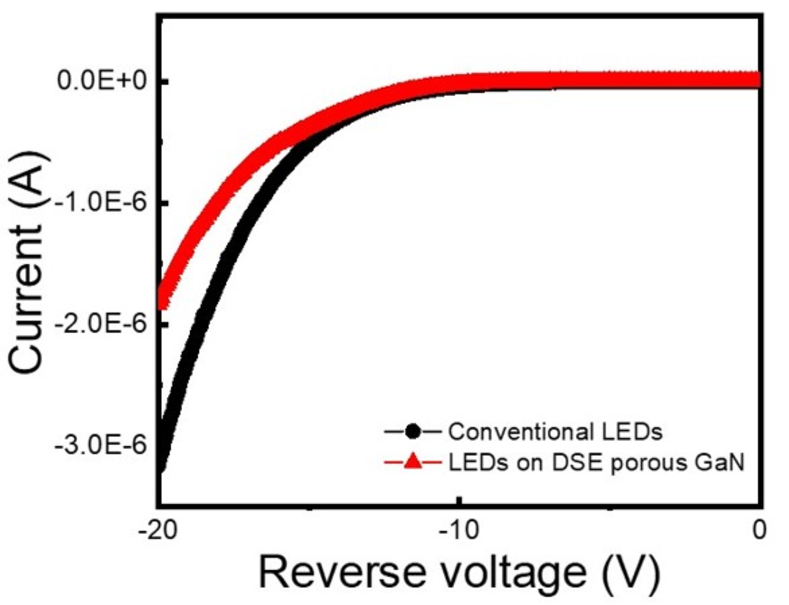

Supplement: S3 Fig — shows the leakage currents of LEDs with and without DSE porous GaN, respectively. The leakage current is estimated to be 2 nA and 80 nA, respectively, for the LEDs grown on DSE porous GaN and conventional GaN, at a reverse voltage of -5V. The observed low reverse bias current can be attributed to the elimination of dislocations by DSE porous GaN. (TIF) [file pone.0277667.s003.tif]
